# Role of molecular adsorbent recirculating system in methotrexate-induced acute liver failure: a case report and literature review

**DOI:** 10.3389/fped.2024.1424919

**Published:** 2024-08-15

**Authors:** T. Corbisier, André O. Von Bueren, W.B. Breunis, S. Grazioli

**Affiliations:** ^1^ Pediatric and Neonatal Intensive Care Unit, Department of Pediatrics, Gynecology and Obstetrics, University Hospital of Geneva, University of Geneva, Geneva, Switzerland; ^2^ Pediatric Oncology and Hematology, Department of Pediatrics, Gynecology and Obstetrics, University Hospital of Geneva, University of Geneva, Zurich, Switzerland; ^3^ Division of Oncology and Hematology, University Children’s Hospital Zurich, Children’s Research Center, Zurich, Switzerland

**Keywords:** methotrexate toxicity, acute liver failure, acute kidney injury, extracorporeal treatment, case report

## Abstract

We describe the case of a 14-year-old girl with osteosarcoma who was treated with high-dose methotrexate (12 g/m^2^). Twenty-four hours after the infusion, her plasma methotrexate concentration was elevated at 937 μmol/L (normal < 10 µmol/L). She exhibited severe signs of methotrexate toxicity, including encephalopathy, acute liver failure (ALF), and acute kidney injury. In this case report, we highlight the severe and rare adverse effects secondary to methotrexate administration and the efficacity of molecular adsorbent recirculating system and continuous venovenous hemodiafiltration to recover from multiple organ failure.

## 1 Introduction

Methotrexate (MTX), a folate antagonist, has a long history in cancer treatment. Initially used in 1948 to treat childhood acute lymphoblastic leukemia (ALL), MTX remains a cornerstone in modern hemato-oncologic therapy for several diseases, including osteosarcoma. The side effects of MTX show significant variations depending on the dosage administered. High-dose methotrexate (HDMTX), defined as a dose higher than 500 mg/m^2^, is associated with multiple potential adverse effects related to the drug dose and duration of drug exposure.

Treatment protocols using HDMTX involve administering a dose of MTX over a 4–36 h infusion period. Frequent therapeutic monitoring is required. Effective rescue treatment necessitates pre- and post-treatment measures like hydration, urinary alkalinization, and multiple doses of leucovorin (1).

Leucovorin, as reduced folates, mitigates the risk of toxicity of high-dose methotrexate by bypassing dihydrofolate reductase to restart the intracellular folate cycle. However, the effect of leucovorin is limited at high methotrexate concentrations (2).

Glucarpidase (recombinant bacterial enzyme carboxypeptidase G2, CPDG2) is a recently approved antidote for methotrexate toxicity (3). It is a recombinant enzyme that hydrolyzes methotrexate to its inactive metabolites [deoxy-4-amino-N10-methylpteroic acid (DAMPA) and glutamic acid], which undergo non-renal elimination via hepatic metabolism. Glucarpidase lowers serum methotrexate concentration by 90%–95% within minutes of administration, with its catalytic effect persisting for 48–72 h (3). Glucarpidase cleaves extracellular methotrexate but has no effect on intracellular methotrexate. This could lead to a rebound in plasma methotrexate concentration from cells in approximately 60% of patients.

The majority of methotrexate is eliminated by the kidneys, proportionally to the degree of renal function. MTX can cause direct renal injury through precipitation in the renal tubules, which could lead to renal failure (2). Only 1%–10% of patients with methotrexate-induced nephrotoxicity require temporary continuous renal replacement therapy (CRRT), with a resolution of acute kidney injury usually occurring after a few months.

The mortality due to HDMTX-induced acute kidney injury is estimated at approximately 42% (4).

About 10%–20% of methotrexate is excreted in bile and metabolized in the liver. Methotrexate is known to cause hepatic fibrosis and cirrhosis in patients receiving long-term treatment with low doses of oral methotrexate, but acute hepatotoxicity after HDMTX is less common (5). Mechanisms of methotrexate-induced hepatotoxicity are multifactorial and include oxidative stress with the generation of reactive oxygen species (ROS), reduction of enzymatic and non-enzymatic antioxidants, induction of a pro-inflammatory response with the release of several inflammatory enzymes and cytokines, and activation of hepatic stellate cells leading to extracellular matrix deposition and hepatic fibrosis (6).

The development of acute liver failure during high-dose methotrexate represents a rare and life-threatening complication that is poorly described in the literature.

Methotrexate, due to its biochemical characteristics, is considered moderately dialyzable. Current scientific recommendations do not endorse the first-line use of extracorporeal treatments for methotrexate intoxication (7). Extracorporeal therapies primarily remove the drug from the plasma compartment, while methotrexate is predominantly present intracellularly. The effectiveness of glucarpidase in rapidly reducing MTX levels is crucial for managing toxic methotrexate plasma concentrations (>1 µmol/L) in adult and pediatric patients with delayed methotrexate clearance. However, extracorporeal therapies provide an essential option, especially in severe cases with renal or hepatic failure.

Here, we describe the case of a 14-year-old girl who developed acute kidney injury and severe acute liver failure following a single high dose of methotrexate. She required organ support with CRRT and a daily session of the molecular adsorbent recirculating system (MARS), in addition to glucarpidase treatment.

## 2 Case description

Our patient is a 14-year-old girl with high-grade osteosarcoma of the right tibia who was treated according to the EURASMOS (European and American Osteosarcoma study) protocol. On day 23 of the protocol, she received a high dose of intravenous methotrexate (12 g/m^2^). Prior to MTX injection, the patient exhibited normal kidney and liver function. According to the protocol, she was hyperhydrated with an alkaline solution and simultaneously received leucovorin.

Despite implementing preventive strategies to limit nephrotoxicity, the patient presented with toxic plasmatic concentrations of methotrexate (as measured using the liquid chromatography–mass spectrometry method); at 4 and 24 h after administration, the methotrexate levels were 1,356 and 937 µmol/L, respectively (the expected methotrexate plasma level at 24  h: <10 µmol/L). On day 1 after the HDMTX injection, the patients started exhibiting clinical signs of severe methotrexate toxicity, including encephalopathy and hemodynamic instability. Laboratory data showed acute liver failure with elevated liver enzymes (ASAT > 7,000 U/L, ALAT 6,570 U/L), severe coagulopathy (INR 2.36, factor V 6.9%), hyperammonemia (123 µmol/L), cholestasis (total bilirubin: 58 µmol/L, direct bilirubin 455 µmol/L), and hyperlactatemia (11.2 mmol/L).

She also exhibited signs of acute kidney injury, with elevated creatinine levels (268 µmol/L) and a decreased glomerular filtration fraction (GFR 32 ml/min/1.73 m^2^) but conserved urine output. Comprehensive additional laboratory investigations (metabolic, toxicological, infectious studies) and systemic anamnesis did not identify any other cause for kidney and liver failure apart from methotrexate intoxication. The history of our patient with a focus on pharmacogenetic explanations has been reported elsewhere (8).

Due to alterations in her level of consciousness and persistent hyperlactatemia, the patient was intubated, and hemodynamic support with vasopressors was started in the context of severe vasoplegia secondary to acute liver failure (ALF).

The patient was also early started on glucarpidase at 36 h (50 U/kg), along with a continuation of nephroprotective measures with leucovorin and hyperhydration with an alkaline solution. Because of her critical condition with multiple organ failure, she was transferred to a liver transplant center.

In the context of severe ALF complicated by persistent multiple organ dysfunction and elevated MTX levels, it was decided to support the patient with liver extracorporeal therapy using the MARS as a bridge to recovery and to enhance methotrexate elimination. Blood and albumin flow rates were set at 120 ml/min, and both dialysis and replacement fluid rates were set between 800 and 1,200 ml/min. Anticoagulation was performed with heparin, adjusted to maintain an activating clotting time between 180 and 200 s. The MARS sessions lasted between 6 and 8 h, and the patient was treated with CRRT in continuous venovenous hemodiafiltration mode (CVVHDF) between the MARS sessions. The CVVHDF was set up on a “Prismaflex” device (Baxter, Deerfield, IL, USA) with the following prescription parameters: pre-filter replacement fluid rate of 1,200 ml/h, post-filter replacement rate of 500 ml/H, fluid removal rate of 50 ml/h, heparin anticoagulation at 10–25 IU/kg/h, and dialysate rate of 900 ml/h.

**Figure 1:**
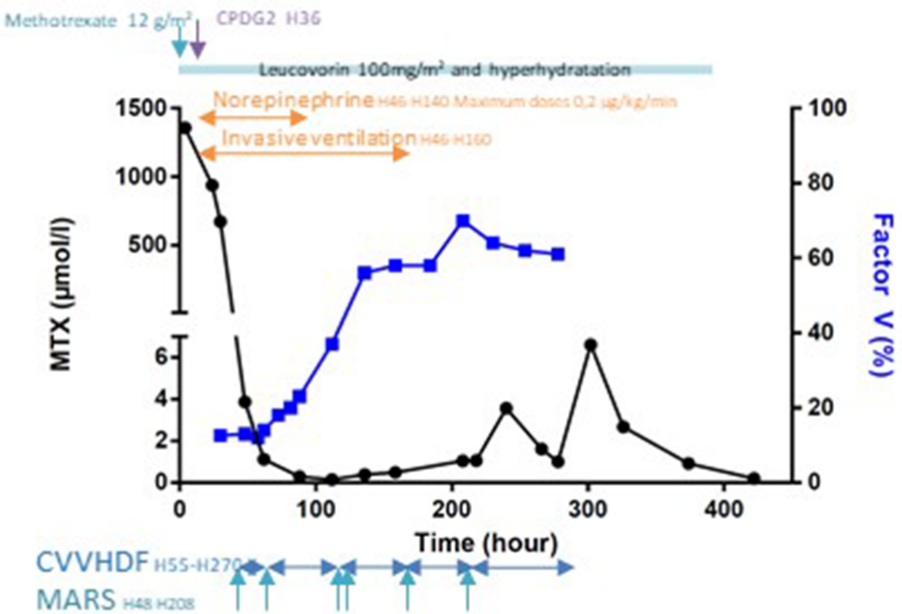
Time course of methotrexate and factor V levels according to extracorporeal therapies with MARS and CRRT.

Over the course of the MARS sessions, the patient's clinical condition began to improve, allowing for a progressive weaning of the hemodynamic and ventilatory support. We could stop vasoactive agents, and she was extubated on day 5 after initiation of extracorporeal therapy (Figure 1). The patient also showed signs of liver regeneration, with factor V levels progressively increasing to 70% by day 9 post-MTX infusion, leading to the arrest of MARS therapy.

The hemodiafiltration was stopped on the same day but had to be resumed the following day due to significant fluid overload despite the administration of high doses of furosemide.

In total, the patient underwent six MARS sessions (160 h) and 9 days of CRRT (215 h).

As expected, blood levels of methotrexate rapidly decreased after the dose of glucarpidase (from 675 to 3.87 µmol/L) and subsequently returned to expected norms within 48 h (9). Ten days after treatment administration, a rebound effect was observed with a measured methotrexate level of 3.56 µmol/L. After the cessation of renal replacement therapy, a subsequent resurgence of methotrexate was observed, albeit without involvement of associated organs, which subsequently diminished with continued leucovorin treatment.

The patient was hospitalized for 11 days in the intensive care unit and was subsequently transferred to the pediatric oncology department. Two months after her hospitalization in the pediatric intensive care unit, she regained normal renal and hepatic function. Unfortunately, the patient's condition progressed, and she was referred to the United States for chimeric antigen receptor (CAR) T-cell therapy.

## 3 Discussion

Although HDMTX is frequently administered to numerous patients, it can cause severe systemic toxicity that may require urgent organ support. This 14-year-old patient required vasopressor support, mechanical invasive ventilation, as well as continuous hemodiafiltration and MARS sessions after the administration of a single high dose of methotrexate, despite administering prophylactic medication treatments.

The use of extracorporeal treatment to reduce methotrexate plasmatic concentrations has been described in the literature. However, the most effective method of MTX removal remains to be established due to heterogeneity in methods and variability in outcomes among the various studies. The physiochemical characteristics of methotrexate are a small molecular weight of 454 Da, a distribution volume of 0.4–0.8 L/kg, and plasma protein binding of 50% (10). In adult patients with HDMTX-induced renal dysfunction, high-flux intermittent hemodialysis resulted in the greatest decrease in plasma MTX concentration (median 76%) within the shortest period compared to hemofiltration, continuous hemodialysis, charcoal hemoperfusion, or plasma exchange (11, 12). Despite this efficacy, our patient was too hemodynamically unstable to tolerate intermittent dialysis and required inotropic support with norepinephrine. The availability of the machine and teams capable of using it did not allow for its use in emergency settings. Continuous hemodialysis is considered a safe alternative, also for pediatric patients (10).

Because of high protein binding of methotrexate, MARS, single-pass albumin dialysis (SPAD), and therapeutic plasma exchange are effective alternatives. Activated charcoal adsorbs methotrexate better than resins, although both are limited by cartridge saturation (7). SPAD does not appear to improve MTX clearance compared with CVVHDF with maximal effluent volumes while adding additional costs (13). Peritoneal dialysis is described as ineffective (10). Plasma exchange appears to be an extracorporeal purification modality of interest because it can remove both protein-bound MTX and non-bound MTX (14). The current increasing interest in plasma exchange for acute liver failure makes it an interesting alternative, particularly in cases involving liver involvement (15).

MARS is an extracorporeal liver support using albumin dialysis. During MARS, blood is pumped countercurrent to a closed-circuit albumin solution, with the contents passing through a charcoal column and anion exchange resin (16). The circulating fluid also runs against conventional continuous venovenous hemofiltration or hemodialysis equipment. This process results in the removal of minimally dialyzable, lipophilic, albumin-bound molecules in addition to the water-soluble products extracted by renal replacement therapies. Methotrexate, due to its biochemical properties, is effectively eliminated by this clearance method, although this is poorly described in the literature. By removing the vasoactive agents (NO and inflammatory cytokines), bilirubin, and bile acids, MARS assists in increasing the systemic vascular resistance, improving hepatic encephalopathy, and reducing bilirubin levels that potentially cause hepatocyte necrosis. Given the possibility of hepatic regeneration, it is more important to promptly initiate clearance treatment to enable recovery from this organ injury with high mortality rates. Unfortunately, Baxter recently issued an end-of-life notification for the MARS 1TC monitor, but there is some suggestion that the combination of CRRT and plasma exchange might offer some of the benefits provided by MARS (17).

The increase in methotrexate concentration after extracorporeal treatments, the so-called rebound effect, averaged 76% compared to pre-sessions (7). This is probably explained by the redistribution of methotrexate from cells and tissues (11). The magnitude of the rebound is greater after more efficient techniques, such as intermittent hemodialysis, making several days of extracorporeal treatment and dosing of methotrexate necessary (7, 18). This redistribution is also seen after glucarpidase injection. In contrast to hemodialysis, cessation of the plasma exchange procedure does not affect the reduction of serum MTX levels (14).

A rebound effect was indeed observed in our patient 240 h after methotrexate administration, with a measured level of 3.56 µmol/L. CVVHDF had been discontinued 6 h prior, highlighting redistribution of the methotrexate from the intracellular to the intravascular space. As methotrexate rapidly distributes into cells and tissues, extracorporeal treatments initiated promptly after methotrexate administration show considerable effectiveness and less significant rebound (7). There would thus be a potential benefit in initiating extracorporeal purification therapy promptly after the diagnosis of methotrexate intoxication and the onset of organ failure.

**Figure 2:**
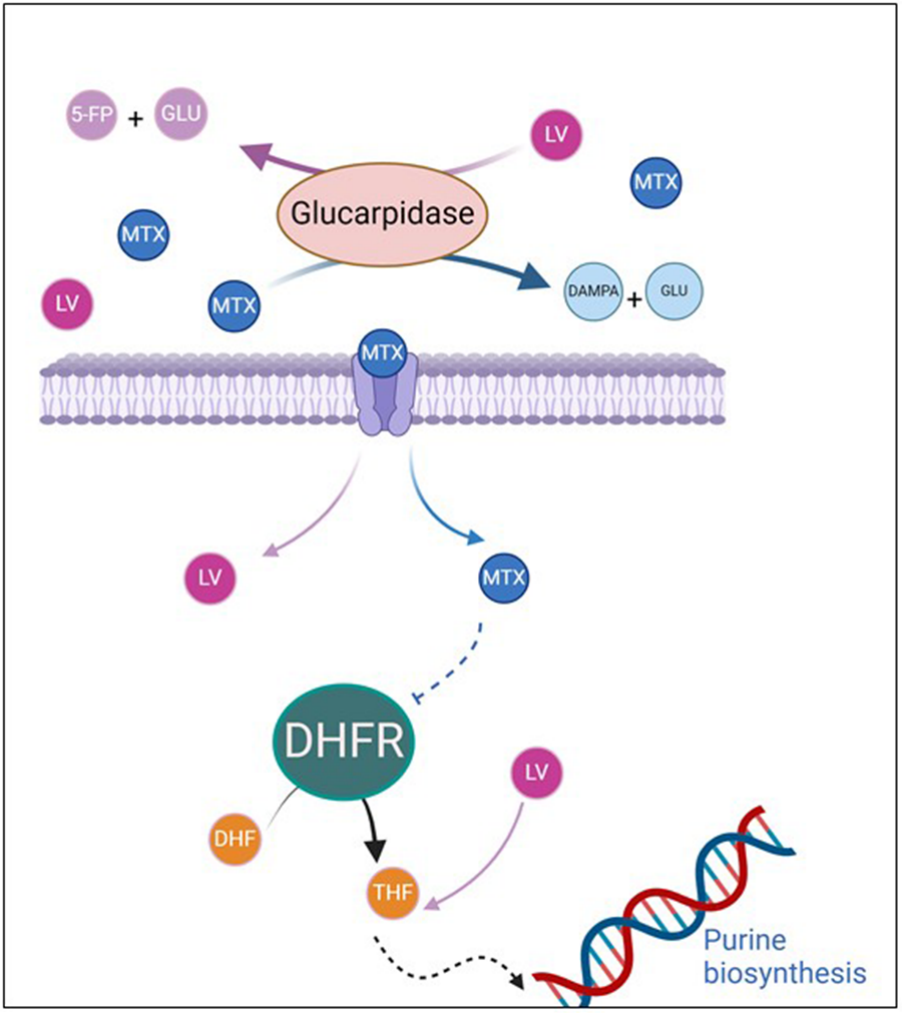
Mechanism of action of glucarpidase and leucovorin in methotrexate toxicity. Methotrexate and leucovorin enter the cell via the RFC receptor and through passive diffusion in cases of high doses. MTX inhibits folate-dependent enzymes, including DHFR, thereby reducing purine production. This leads to a decrease in intracellular folate and inhibition of DNA synthesis. Leucovorin is essential for restoring sufficient intracellular folate levels. Glucarpidase rapidly reduces methotrexate levels by cleaving it into two inactive metabolites (DAMPA and glutamic acid), both of which are eliminated by the liver rather than the kidneys. Glucarpidase also inactivates leucovorin (5-FP and glutamic acid). 5FP, 5-formylpteroate; DAMPA, 4-deoxy-4-amino-N10-methylpteroic acid; DHFR, dihydrofolate reductase; GLU, glutamate; LV, leucovorin; MTX, methotrexate; RFC, reduced folate carrier; THE, tetrahydrofolate.

The recent use of glucarpidase (Figure 2) in the context of renal failure due to methotrexate intoxication allows a very rapid decrease in blood methotrexate levels (19). The inactivated metabolite of methotrexate by glucarpidase is next preferentially eliminated by the liver, which justifies extracorporeal liver support in our patient with acute liver failure (7). Glucarpidase cleaves intravascular methotrexate but has no effect on intracellular methotrexate, where MTX exerts its efficacity and toxicity. Despite this rapid decrease in methotrexate levels, the severity of the involvement of both liver and kidney organs led us to initiate CVVHDF and MARS to support both organs.

Leucovorin is cleared by extracorporeal treatment and hydrolyzed by leucovorin (7). Therefore, it is recommended to continue this treatment until therapeutic levels are achieved (10).

Particular emphasis should be placed on therapeutic monitoring, as methotrexate concentrations cannot be monitored by immunoassays up to 48 h after glucarpidase use due to cross reactivity with its metabolites (7). Specific dosage by liquid chromatography–mass spectrometry (LC–MS) needs to be performed because this technique can reliably differentiate MTX concentrations from its metabolites. These methods may not be available at many centers. In our case, the immunoassay-based dosage performed at 48 h of methotrexate administration showed a falsely elevated level of 900 µmol/L, while the specific high performance liquid chromatography (HPLC)-based assay measured it at 3.87 µmol/L.

It is challenging to determine the best extracorporeal therapies for methotrexate clearance because of the inherent complexity of methotrexate pharmacokinetics. Intermittent hemodialysis is probably the most efficient extracorporeal treatment, but it requires hemodynamic stability (7). Continuous hemodiafiltration seems to be more efficient than MARS in our patient, as suggested by the rebound effect at the cessation of CVVHDF and the already hemodynamic improvement before the first session of MARS. In this case, MARS was utilized in the context of hepatocellular insufficiency present in our patient. For renal insufficiency alone due to methotrexate intoxication, high-flow CVVHDF would have been used exclusively.

## 4 Conclusions

This case report highlights the severe and life-threatening complications that can arise from high-dose methotrexate administration, including acute liver failure and acute kidney injury. The utilization of extracorporeal treatments such as CRRT and MARS proved to be effective in managing these complications and facilitating the recovery of renal and hepatic function in our patient. Despite the challenges associated with high-dose methotrexate toxicity, advancements such as glucarpidase therapy offer promising avenues for the rapid reduction of methotrexate levels. However, the rebound effect observed underscores the importance of vigilant monitoring and the need for continued supportive therapies.

The clinician's decision should be guided by the therapies available in their department and the patient's clinical condition and potentially consider combining two clearance modalities (for example, plasmapheresis or MARS along with hemodialysis).

Further research is warranted to establish optimal treatment strategies and improve outcomes in patients with methotrexate-induced acute liver failure.

## Data Availability

The raw data supporting the conclusions of this article will be made available by the authors without undue reservation.

## References

[1] Ackland SP Schilsky RL. High-dose methotrexate: a critical reappraisal. J Clin Oncol. (1987) 5(12):2017–31. 10.1200/JCO.1987.5.12.2017 3316519

[2] Howard SC McCormick J Pui CH Buddington RK Harvey RD. Preventing and managing toxicities of high-dose methotrexate. Oncologist. (2016) 21(12):1471–82. 10.1634/theoncologist.2015-0164 27496039 PMC5153332

[3] Ramsey LB Balis FM O’Brien MM Schmiegelow K Pauley JL Bleyer A Consensus guideline for use of glucarpidase in patients with high-dose methotrexate induced acute kidney injury and delayed methotrexate clearance. Oncologist. (2018) 23(1):52–61. 10.1634/theoncologist.2017-0243 29079637 PMC5759822

[4] Gros L Roldán A Cabero-Martínez A Domínguez-Pinilla N de la Fuente A González-Barca E Incidence and management of patients with methotrexate delayed elimination in the clinical practice: a Delphi study. J Oncol Pharm Pract. (2023) 29(4):794–801. 10.1177/10781552221079568 35147457 PMC10273868

[5] Kremer JM Alarcón GS Lightfoot RW Willkens RF Furst DE Williams HJ Methotrexate for rheumatoid arthritis. Suggested guidelines for monitoring liver toxicity. American College of Rheumatology. Arthritis Rheum. (1994) 37(3):316–28. 10.1002/art.1780370304 8129787

[6] Ezhilarasan D. Hepatotoxic potentials of methotrexate: understanding the possible toxicological molecular mechanisms. Toxicology. (2021) 458:152840. 10.1016/j.tox.2021.152840 34175381

[7] Ghannoum M Roberts DM Goldfarb DS Heldrup J Anseeuw K Galvao TF Extracorporeal treatment for methotrexate poisoning: systematic review and recommendations from the EXTRIP workgroup. Clin J Am Soc Nephrol. (2022) 17(4):602–22. 10.2215/CJN.08030621 35236714 PMC8993465

[8] El Masri AER Tobler C Willemijn B Von Bueren AO Ansari M Samer CF. Case report: hepatotoxicity and nephrotoxicity induced by methotrexate in a paediatric patient, what is the role of precision medicine in 2023? Front Pharmacol. (2023) 14:1130548. 10.3389/fphar.2023.1130548 37201023 PMC10185764

[9] Holmboe L Andersen AM Mørkrid L Slørdal L Hall KS. High dose methotrexate chemotherapy: pharmacokinetics, folate and toxicity in osteosarcoma patients. Br J Clin Pharmacol. (2012) 73(1):106–14. 10.1111/j.1365-2125.2011.04054.x 21707700 PMC3248260

[10] Raina R Grewal MK Blackford M Symons JM Somers MJG Licht C Renal replacement therapy in the management of intoxications in children: recommendations from the pediatric continuous renal replacement therapy (PCRRT) workgroup. Pediatr Nephrol. (2019) 34(11):2427–48. 10.1007/s00467-019-04319-2 31446483

[11] Widemann BC Balis FM Kempf-Bielack B Bielack S Pratt CB Ferrari S High-dose methotrexate-induced nephrotoxicity in patients with osteosarcoma. Cancer. (2004) 100(10):2222–32. 10.1002/cncr.20255 15139068

[12] Wall SM Johansen MJ Molony DA DuBose TD Jaffe N Madden T. Effective clearance of methotrexate using high-flux hemodialysis membranes. Am J Kidney Dis. (1996) 28(6):846–54. 10.1016/S0272-6386(96)90384-4 8957036

[13] Vilay AM Mueller BA Haines H Alten JA Askenazi DJ. Treatment of methotrexate intoxication with various modalities of continuous extracorporeal therapy and glucarpidase. Pharmacotherapy. (2010) 30(1):111. 10.1592/phco.30.1.111 20030480

[14] Cecyn KZ Lee J Oguro T Petrilli AS Bordin JO. Use of plasma exchange in methotrexate removal in a patient with osteosarcoma and acute renal insufficiency. Am J Hematol. (2003) 72(3):209–11. 10.1002/ajh.10271 12605394

[15] Zachariah U Vijayalekshmi B Matthai SM Goel A Eapen CE. Extra-corporeal non-liver transplant therapies for acute liver failure: focus on plasma exchange and continuous renal replacement therapy. Indian J Gastroenterol. (2024) 43(2):338–48. 10.1007/s12664-024-01558-6 38530631

[16] Baker DR Mac H Steinman B Soshnick SH Frager SZ Goilav B Molecular adsorbent recirculating system for acute liver failure in a new pediatric-based extracorporeal liver support program. Crit Care Explor. (2023) 5(11):e1002. 10.1097/CCE.0000000000001002 37954902 PMC10635609

[17] Schaefer B Schaefer F Engelmann G Meyburg J Heckert KH Zorn M Comparison of molecular adsorbents recirculating system (MARS) dialysis with combined plasma exchange and haemodialysis in children with acute liver failure. Nephrol Dial Transplant. (2011) 26(11):3633–9. 10.1093/ndt/gfr115 21421589

[18] Kitamura M Kitamura S Fujioka M Kamijo R Sato S Sawayama Y Methotrexate-induced acute kidney injury in patients with hematological malignancies: three case reports with literature review. Ren Replace Ther. (2018) 4(1):39. 10.1186/s41100-018-0180-9

[19] Kielbowski K Rosik J Bakinowska E Gromowska E Ustianowski Ł Szostak B The use of glucarpidase as a rescue therapy for high dose methotrexate toxicity—a review of pharmacological and clinical data. Expert Opin Drug Metab Toxicol. (2023) 19(11):741–50. 10.1080/17425255.2023.2272593 37846862

