# Supplementary material for: Role of molecular adsorbent recirculating system in methotrexate-induced acute liver failure: a case report and literature review
Source: Front Pediatr. 2024 Aug 15;12:1424919. doi: 10.3389/fped.2024.1424919 (PMC11363709; doi:10.3389/fped.2024.1424919)
Supplement: Supplementary file 1 [file Datasheet1.pdf]

		Normal range	H 4	H 24	H 30	H 48	H 62	H 88	H 120	H 136	H 208	H 218	H 240	H 266	H 278	H 302	H 326	H 374	H 422	H 878
**Urea**	**mmol/L**	**3.2-7.5**		10,4	11,1	11,7	8,5	3,2	2,3	2,2	2	6,6	6,7	3,2	3,1	5,4	7,1	7,6	6,3	2,9
**Creatininemia**	**µmol/l**	**37-69**		286	321	340	180	144	129	114	101	316	293	173	173	291	321	218	123	50
**Methotrexate**	**µmol/l**		1356	937	672	3	1,1	0,2	0,1	0,3	1,0	1,0	3,5	1,6	1	6,6	2,6	0,9	0,2	

Table 1 :

The temporal evolution of blood methotrexate, creatinine, and urea levels. H0 is defined as the time of high-dose methotrexate administration
