# Supplementary figures and images for: Role of molecular adsorbent recirculating system in methotrexate-induced acute liver failure: a case report and literature review
Source: Front Pediatr. 2024 Aug 15;12:1424919. doi: 10.3389/fped.2024.1424919 (PMC11363709; doi:10.3389/fped.2024.1424919)

## Bili-creat

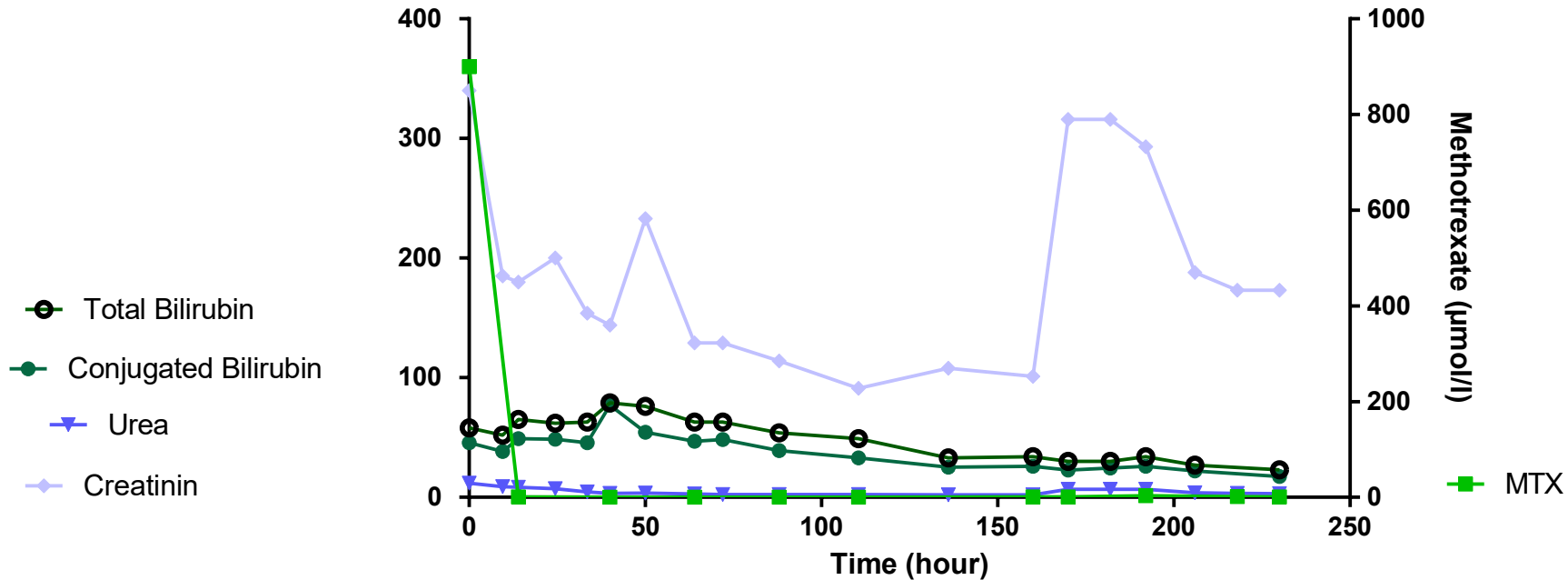

Supplement: Supplementary file 2 [file Datasheet2.pdf]
